# Supplementary material for: Training Together, Diagnosing Better: Federated Learning for Collagen VI–Related Dystrophies
Source: ArXiv. 2025 Dec 18:arXiv:2512.16876v1. Preprint. [Version 1] (PMC13175213)
Supplement: Supplement 1 [file NIHPP2512.16876v1-supplement-1.pdf]

9 Supplementary material

This section describes the study population's genetic and demographic characteristics, as well as the mathematical formulation of the ML and FL models.

A Genetic and demographic characteristics

Table S1: Detailed genetic and demographic characteristics of the study population for NIH.

**Patient ID**	**# images**	**Sex**	**Age at biopsy (Age at lab sample transfer) (y.o.)**	**Gene**	**Mutation**	**Protein Change**	**Pathogenic mechanism**
NIH-1	16	F	39	COL6A1	c.877G>A	G293R	Gly substitution
NIH-2	6	M	(11)	COL6A1	c.1056+1G>A	Ex14skip	Exon skipping
NIH-3	8	F	17	COL6A1	c.930+189C>T	Intron11 pseudoexon	Pseudoexon insertion
NIH-4	4	M	16	COL6A1	c.868G>A	G290R	Gly substitution
NIH-5	5	F	(11)	COL6A3	c.6210+1G>A	Ex16skip	Exon skipping
NIH-7	15	M	18	COL6A1	c.877G>A	G293R	Gly substitution
NIH-8	15	M	(7)	COL6A1	c.877G>A	G293R	Gly substitution
NIH-9	39	N/A	N/A	Control	N/A	N/A	Control

Continued on next page

Table S1: Detailed genetic and demographic characteristics of the study population for NIH. (Continued)

Patient ID	# images	Sex	Age at biopsy (Age at lab sample transfer) (y.o.)	Gene	Mutation	Protein Change	Pathogenic mechanism
NIH-10	16	M	N/A	Control	N/A	N/A	Control
NIH-11	6	F	30	Control	N/A	N/A	Control
NIH-13	4	M	3	Control	N/A	N/A	Control
NIH-14	7	M	(2)	COL6A2	c.812G>A	G271D	Gly substitution
NIH-15	7	F	(10)	COL6A1	c.930+189C>T	Intron11 pseudoexon	Pseudoexon insertion
NIH-16	6	M	12	COL6A1	c.930+189C>T	Intron11 pseudoexon	Pseudoexon insertion
NIH-17	6	F	18	COL6A1	c.930+189C>T	Intron11 pseudoexon	Pseudoexon insertion
NIH-18	7	F	8	COL6A1	c.930+189C>T	Intron11 pseudoexon	Pseudoexon insertion
NIH-19	6	F	24	Control	N/A	N/A	Control
NIH-20	7	F	(9)	Control	N/A	N/A	Control
NIH-21	6	N/A	N/A	Control	N/A	N/A	Control

Continued on next page

Table S1: Detailed genetic and demographic characteristics of the study population for NIH. (Continued)

Patient ID	# images	Sex	Age at biopsy (Age at lab sample transfer) (y.o.)	Gene	Mutation	Protein Change	Pathogenic mechanism
NIH-22	8	F	(3)	COL6A1	c.930+189C>T	Intron11 pseudoexon	Pseudoexon insertion
NIH-23	6	F	(5)	COL6A1	c.904-3T>G	Ex11skip	Exon skipping
NIH-24	6	F	5	COL6A1	c.1056+1G>A	Ex14skip	Exon skipping
NIH-25	6	F	(22)	COL6A3	c.6210+1G>A	Ex16skip	Exon skipping
NIH-26	1	M	9	COL6A3	c.6309+3A>G	Ex18skip	Exon skipping
NIH-27	2	M	15	COL6A1	c.930+189C>T	Intron11 pseudoexon	Pseudoexon insertion
NIH-28	1	M	N/A	COL6A1	N/A	Ex14skip	Exon skipping
NIH-29	1	M	N/A	COL6A3	c.6156+1G>A	Ex15skip	Exon skipping
NIH-30	1	N/A	N/A	COL6A1	c.850G>A	G284R	Gly substitution
NIH-31	1	F	(35)	COL6A1	c.1056+1G>A	Ex14skip	Exon skipping

Continued on next page

Table S1: Detailed genetic and demographic characteristics of the study population for NIH. (Continued)

Patient ID	# images	Sex	Age at biopsy (Age at lab sample transfer) (y.o.)	Gene	Mutation	Protein Change	Pathogenic mechanism
NIH-32	2	F	(12)	COL6A1	c.815G>A	G272D	Gly substitution
NIH-33	2	M	(9)	COL6A3	c.6210+5G>A	Ex16skip	Exon skipping
NIH-34	2	M	(3)	COL6A1	c.904-10G>A	Ex11skip	Exon skipping
NIH-35	1	F	(40)	COL6A1	c.859G>A	G287R	Gly substitution
NIH-36	1	M	(15)	COL6A1	c.850G>A	G284R	Gly substitution
NIH-37	2	F	26	COL6A3	c.6229G>C	G2077R	Gly substitution
NIH-38	2	F	(3)	COL6A1	c.904-1G>C	Ex11skip	Exon skipping
NIH-39	2	M	(2)	COL6A3	c.6210+1G>A	Ex16skip	Exon skipping
NIH-40	2	M	(14)	COL6A1	c.805-2A>G	Ex9skip	Exon skipping
NIH-41	2	F	(3)	COL6A1	c.842G>A	G281E	Gly substitution

Continued on next page

Table S1: Detailed genetic and demographic characteristics of the study population for NIH. (Continued)

Patient ID	# images	Sex	Age at biopsy (Age at lab sample transfer) (y.o.)	Gene	Mutation	Protein Change	Pathogenic mechanism
NIH-42	2	M	(8)	COL6A2	c.1333-1G>A	Ex16skip	Exon skipping
NIH-43	2	M	(7)	COL6A3	c.6210+5insA	Ex16skip	Exon skipping
NIH-44	2	M	3	COL6A3	c.6156+1G>A	Ex15skip	Exon skipping
NIH-45	2	F	22	COL6A1	c.930+189C>T	Intron 11 pseudoexon	Pseudoexon insertion
NIH-46	2	M	(11)	COL6A3	c.6210+1G>A	Ex16skip	Exon skipping
NIH-47	2	F	(12)	COL6A1	c.823G>A	G275R	Gly substitution
NIH-48	2	F	(18)	COL6A1	c.1056+1G>T	Ex14skip	Exon skipping
NIH-49	2	M	12	COL6A3	c.6221G>A	G2074D	Gly substitution
NIH-50	2	M	(14)	COL6A1	c.1003-2A>G	Ex14skip	Exon skipping
NIH-51	2	F	30	COL6A1	c.805-641_858+276del	Ex9skip	Exon skipping

Continued on next page

Table S1: Detailed genetic and demographic characteristics of the study population for NIH. (Continued)

Patient ID	# images	Sex	Age at biopsy (Age at lab sample transfer) (y.o.)	Gene	Mutation	Protein Change	Pathogenic mechanism
NIH-52	2	M	(14)	COL6A1	c.841G>A	G281R	Gly substitution
NIH-53	2	M	(7)	COL6A1	c.904G>A	G302R	Gly substitution
NIH-54	2	M	(6)	COL6A1	c.930+189C>T	Intron 11 pseudoexon	Pseudoexon insertion
NIH-56	2	F	(12)	COL6A1	c.868G>A	G290R	Gly substitution
NIH-57	2	M	18	COL6A1	c.1184G>T	G395V	Gly substitution
NIH-58	1	M	7	COL6A1	c.868G>A	G290R	Gly substitution
NIH-60	2	M	(N/A)	COL6A1	c.850G>A	G284R	Gly substitution
NIH-61	2	F	14	COL6A3	c.6248G>A	G2083D	Gly substitution
NIH-62	2	F	2	COL6A2	c.901-3_914del17bp	Ex8skip	Exon skipping
NIH-63	2	F	(23)	COL6A1	c.930+189C>T	Intron 11 pseudoexon	Pseudoexon insertion

Continued on next page

Table S1: Detailed genetic and demographic characteristics of the study population for NIH. (Continued)

Patient ID	# images	Sex	Age at biopsy (Age at lab sample transfer) (y.o.)	Gene	Mutation	Protein Change	Pathogenic mechanism
NIH-64	2	F	(6)	COL6A3	c.6157G>T	G2053C	Gly substitution
NIH-65	2	M	(N/A)	COL6A3	c.6308A>G	Ex18skip	Exon skipping
NIH-66	1	N/A	7	COL6A2	c.855+1G>A	Ex6skip	Exon skipping
NIH-67	2	M	55	COL6A1	c.1056+1G>A	Ex14skip	Exon skipping
NIH-68	2	M	21	COL6A1	c.868G>A	G290R	Gly substitution
NIH-69	1	F	(12)	COL6A1	c.930+189C>T	Intron 11 pseudoexon	Pseudoexon insertion
NIH-70	1	M	(42)	COL6A1	N/A	Ex14skip	Exon skipping
NIH-72	1	F	9	COL6A1	N/A	Ex14skip	Exon skipping
NIH-74	1	M	3	COL6A3	c.6309G>C	Ex18skip	Exon skipping
NIH-75	1	F	14	COL6A1	c.850G>A	G284R	Gly substitution

Continued on next page

Table S1: Detailed genetic and demographic characteristics of the study population for NIH. (Continued)

Patient ID	# images	Sex	Age at biopsy (Age at lab sample transfer) (y.o.)	Gene	Mutation	Protein Change	Pathogenic mechanism
NIH-76	1	M	7	COL6A3	c.6248G>A	G2083D	Gly substitution
NIH-77	2	M	0	COL6A1	c.850G>A	G284R	Gly substitution
NIH-78	1	M	(N/A)	COL6A2	c.820G>A	G274S	Gly substitution
NIH-79	2	F	(3)	COL6A2	c.1162G>A	G388R	Gly substitution
NIH-80	2	M	3	COL6A2	c.954G>A	Ex9skip	Exon skipping
NIH-81	2	N/A	N/A	COL6A1	c.956A>G	K319R	Exon skipping

Table S2: Detailed genetic and demographic characteristics of the study population for UCL.

Patient ID	# images	Sex	Age at biopsy (y.o.)	Gene	Mutation	Protein Change	Pathogenic mechanism
UCL-1	2	F	23	COL6A1	c.930+189C>T	Intron11pseudoexon	Pseudoexon insertion
UCL-2	2	M	3	COL6A1	c.930+189C>T	Intron11pseudoexon	Pseudoexon insertion

Continued on next page

Table S2: Detailed genetic and demographic characteristics of the study population for UCL. (Continued)

Patient ID	# images	Sex	Age at biopsy (y.o.)	Gene	Mutation	Protein Change	Pathogenic mechanism
UCL-3	3	M	7	COL6A1	c.930+189C>T	Intron11pseudoexon	Pseudoexon insertion
UCL-4	1	M	6	COL6A1	c.930+189C>T	Intron11pseudoexon	Pseudoexon insertion
UCL-5	4	M	N/A	COL6A1	c.1056+1G>A	Ex14skip	Exon skipping
UCL-6	1	M	12	COL6A1	c.1776+1G>A	Ex27skip	Exon skipping
UCL-7	1	F	22	COL6A2	c.2839_2850del	p.Leu947_Gly950del	Deletion
UCL-8	1	N/A	N/A	COL6A1	c.428+1G>A	Ex3skip	Exon skipping
UCL-9	3	F	3	COL6A3	c.6309+3A>G	Ex18skip	Exon skipping
UCL-10	1	M	N/A	COL6A2	c.2839_2850del	p.Leu947_Gly950del	Deletion
UCL-11	1	F	29	COL6A1	c.1022G>T	G341V	Gly substitution
UCL-12	2	M	33	COL6A1	c.877G>A	G293R	Gly substitution
UCL-13	1	N/A	N/A	COL6A1	c.868G>A	G290R	Gly substitution
UCL-14	1	N/A	N/A	COL6A1	c.868G>A	G290R	Gly substitution
UCL-15	7	F	54	Control	N/A	N/A	Control

Table S3: Sex distribution and age statistics per site.

Site ID	SEX			AGE: Male				AGE: Female			
	M	F	N/A	Average	Std. Dev.	Min	Max	Average	Std. Dev.	Min	Max
**NIH**	38	32	5	11.7	11.0	0	55	15.5	10.7	2	40
**UCL**	7	5	3	12.2	10.8	3	33	19.3	9.8	3	29

B ML approach

Given an input space $\mathcal{X} := \mathbb{R}^d$ and output set $\mathcal{Y} \subseteq \mathbb{R}^m$, the goal of Supervised ML is roughly to approximate an unknown function

$$f : \mathcal{X} \longrightarrow \mathcal{Y}, \tag{6}$$

given a dataset $\mathcal{D} = \{(\vec{x}^i, \vec{y}^i)\}_{i=1}^N \subset \mathcal{X} \times \mathcal{Y}$, composed of N known but possibly noisy examples, i.e.

$$\vec{y}^i \simeq f(\vec{x}^i). \tag{7}$$

This approximation problem is typically formulated as the minimization of an Empirical Risk (ER)[37,38] on some training data.

To that purpose (together with preprocessing), the dataset

$$\mathcal{D} = \left\{ \left(\vec{x}^i, \vec{y}^i\right) \right\}_{i=1}^N \tag{8}$$

is firstly split into

a) Training data

$$\mathcal{D}_{\text{train}} = \left\{ \left(\vec{x}^i, \vec{y}^i\right) \right\}_{i \in I_{\text{train}}}; \tag{9}$$

b) Testing data

$$\mathcal{D}_{\text{test}} = \left\{ \left(\vec{x}^i, \vec{y}^i\right) \right\}_{i \in I_{\text{test}}}; \tag{10}$$

c) Validation data

$$\mathcal{D}_{\text{validation}} = \left\{ \left(\vec{x}^i, \vec{y}^i\right) \right\}_{i \in I_{\text{validation}}}, \tag{11}$$

with

$$\{1,\dots,N\} = I_{\text{train}} \bigsqcup I_{\text{test}} \bigsqcup I_{\text{validation}}. \tag{12}$$

At this point, for example, the empirical risk can be defined as

$$J : \boldsymbol{\Theta} \longrightarrow \mathbb{R} \tag{13}$$

$$J(\boldsymbol{\theta}) := \frac{1}{|I_{\text{train}}|} \sum_{i \in I_{\text{train}}} \text{loss}\Big(\mathsf{f}_{\boldsymbol{\theta}}(\vec{x}^{\,i}), \vec{y}^{\,i}\Big) + \lambda \text{Reg}\,(\boldsymbol{\theta})\,, \tag{14}$$

where

- The parameters space $\boldsymbol{\Theta}$ is a Hilbert space on $\mathbb{R}$;
- The continuous loss function

  $$\text{loss} : \mathbb{R}^m \times \mathcal{Y} \longrightarrow \mathbb{R}^+$$

  penalizes the mismatch between the predictions $\mathsf{f}_{\boldsymbol{\theta}}(\vec{x}^{\,i})$ and the labels $\vec{y}^{\,i}$;
- The *regularization* term $\lambda\text{Reg}\,(\boldsymbol{\theta})$ penalizes the model overfitting on training data, the effect of this penalization being modulated by the weighting factor $\lambda > 0$ and $\text{Reg} : \boldsymbol{\Theta} \longrightarrow \mathbb{R}^+$ being a function (e.g., $\text{Reg}\,(\boldsymbol{\theta}) = \|\boldsymbol{\theta}\|^2_{\boldsymbol{\Theta}}$ the squared Hilbertian norm);
- The model

  $$\mathsf{f}_{\boldsymbol{\theta}} : \mathbb{R}^d \longrightarrow \mathbb{R}^m \tag{15}$$

  is a function, belonging to a class

  $$\mathscr{C} = \big\{\mathsf{f}_{\boldsymbol{\theta}} \mid \boldsymbol{\theta} \in \boldsymbol{\Theta}\big\},$$

  $\boldsymbol{\theta}$ being the so-called trainable parameters; examples of $\mathscr{C}$ are Deep Neural Networks[38], Random Forests[39], Gradient Boosting Decision Trees[40], Transformers[41], Large Language Models[42] and Residual Neural Networks (ResNets)[43]; $\mathsf{f}_{\boldsymbol{\theta}}$ is designed to approximate (6), for an appropriate choice of the parameters $\boldsymbol{\theta}$.

In the above context, the ML training is reduced to an *empirical risk minimization problem*,

formulated as

$$\boldsymbol{\theta}^* \in \underset{\boldsymbol{\Theta}}{\text{argmin}} J(\boldsymbol{\theta}). \tag{16}$$

Remark 1 (Existence of solutions)**.** *Existence of a solution to* (16) *might be analysed by the Direct Method in the Calculus of Variations*[44]*.*

For instance, existence holds, assuming

- *The parameters space $\boldsymbol{\Theta}$ is finite dimensional;*
- *The regularisation weighting parameter $\lambda > 0$;*
- *The regularisation function is the Hilbertian norm*

$$\textit{Reg}\,(\boldsymbol{\theta}) = \|\boldsymbol{\theta}\|^2_{\boldsymbol{\Theta}}\,; \tag{17}$$

- *For any $\vec{x} \in \mathbb{R}^d$, the function*

$$\boldsymbol{\theta} \mapsto f_{\boldsymbol{\theta}}(\vec{x}) \tag{18}$$

is continuous.

Remark 2 (Convexity)**.** *J might not be convex, even if loss is convex. Indeed, convexity also depends on*

$$\boldsymbol{\theta} \mapsto f_{\boldsymbol{\theta}}. \tag{19}$$

In case J is not strictly convex, even if a global minimiser exists, its uniqueness is not guaranteed.

The subscript $\boldsymbol{\theta}$ is typically dropped to simplify the notation.

C FL and Horizontal FL

The collaborative network of the present study spans two countries, with data from two distinct sites. In this section, we begin with the setting we defined for ML and extend it to FL.

Consider a dataset $\mathcal{D} = \{(\vec{x}^i, \vec{y}^i)\}_{i=1}^N \subset X \times Y$ composed of N known but possible noisy examples and a federation made of P parties $\{F_k\}_{k=1}^P$.

Suppose the examples of $\mathcal{D}$ are distributed among parties, i.e, party F_k owns the k-th dataset

$$(20)$$

where $N = \bigsqcup_{k=1}^P I_k$ where the symbol $\bigsqcup$ represents a disjoint union. Therefore, in this context, the dataset $\mathcal{D}$ is partitioned as follows

$$\mathcal{D}_k := \{(\vec{x}^i, \vec{y}^i) \mid i \in I_k\}\mathcal{D} = \bigsqcup_{k=1}^P \mathcal{D}_k \tag{21}$$

, which can be seen as a horizontal slicing. The dataset $\mathcal{D}$ is defined as a table where the examples, that is, the images, are its rows.

In this context, our goal is approximating an *unknown function*$f : \mathcal{X} \longrightarrow \mathcal{Y}$ from the N known but possibly noisy examples $\{(\vec{x}^i, \vec{y}^i)\}_{i=1}^N \subset X \times Y$ dispersed across parties as in 21.

In a standard Supervised ML scenario, this is often reduced to an *empirical risk minimization*, as described in Section 4.3.

In this horizontal scenario, the examples $(\vec{x}^i, \vec{y}^i)$ are dispersed across parties, whence no party can fully evaluate the functional defined in 14. However, we can rewrite the function as the average of functions that can be handled by each party

$$J(\boldsymbol{\theta}) = \sum_{k=1}^P \lambda_k J_k(\boldsymbol{\theta}) \tag{22}$$

where $N_k := |I_k|$ is the number of examples possessed by party F_k and the weighting parameter $\lambda_k := \frac{N_k}{N}$ and

$$J_k(\boldsymbol{\theta}) := \frac{1}{N_k} \sum_{i \in I_k} \mathrm{loss}\Big(\mathsf{f}_{\boldsymbol{\theta}}(\vec{x}^i), \vec{y}^i\Big) + \frac{\alpha N}{N_k P} ||\boldsymbol{\theta}||_{\Theta}^2 \tag{23}$$

Note that, if $N_k = \frac{N}{P}$ for every $k \in ||P||$, we have $\frac{\alpha N}{N_k P} = \alpha$.

Hence, in a federated setting, the minimization 14 can be reduced to

a) Minimization of J_k, which is a local task assigned to party F_k

b) Aggregation of the local results

The problem of Horizontal FL is to interlace a) and b) to have an optimal trade-off between privacy, predictive performance, and overhead of communications.
